# Subpial dissection technique in endoscopic endonasal surgery for craniopharyngiomas with supradiaphragmatic extension

**DOI:** 10.3171/2026.7.FOCVID2685

**Published:** 2026-10-01

**Authors:** Takeshi Hongo, Yusuke Morinaga, Shunsuke Shibao, Kei Iwamoto, Sotaro Oshida, Takashi Kashiwagi, Yasuhiro Tsunemi, Tsuguhisa Nakayama, Tetsuya Yamamoto, Hiroyoshi Akutsu

**Affiliations:** 1 Department of Neurosurgery, Yokohama City University Graduate School of Medicine, Yokohama, Kanagawa;; 2 Department of Neurosurgery, Dokkyo Medical University, Mibu, Tochigi;; 3 Department of Neurosurgery, Iwate Medical University, Yahaba, Iwate; and; 4 Department of Otorhinolaryngology-Head and Neck Surgery, Dokkyo Medical University, Mibu, Tochigi, Japan

**Keywords:** craniopharyngioma, endoscopic endonasal surgery, skull base, hypothalamic preservation, pediatric tumor, minimally invasive surgery

## Abstract

Craniopharyngioma surgery requires balancing maximal resection with functional preservation, particularly in supradiaphragmatic tumors. We demonstrate a subpial dissection technique during an endoscopic endonasal approach for a transinfundibular-to-retroinfundibular craniopharyngioma in a 4-year-old girl. Based on the concept that the tumor surface is circumferentially covered by a continuous pial layer extending from the pituitary stalk, dissection was performed within the subpial plane to achieve radical tumor removal while maximizing preservation of hypothalamic and pituitary function. This technique avoids direct manipulation of critical neurovascular structures, facilitates preservation of the endocrine function, and can be applied across various growth patterns of suprasellar craniopharyngiomas.

The video can be found here: https://stream.cadmore.media/r10.3171/2026.7.FOCVID2685

## Transcript

### 0:24 Introduction.

Craniopharyngioma surgery requires balancing maximal tumor removal with preservation of hypothalamic, pituitary, and neurovascular function.^1,2^ Considering the site of origin, the tumor surface is thought to be circumferentially covered by a continuous pial layer extending from the pituitary stalk, providing a natural surgical plane.^3^ In this video, we demonstrate the subpial dissection technique through an endoscopic endonasal approach for radical removal while maximizing hypothalamic and pituitary preservation. The patient is a 4-year-old girl with headache and lethargy. Over several weeks, she developed progressive headache, dizziness, apathy, and slowed responsiveness. Initial MRI showed a cystic enhancing suprasellar lesion with obstructive hydrocephalus. Emergency endoscopic transventricular cyst fenestration and Ommaya reservoir placement were performed. Preoperative basal hormone levels showed preserved thyroid function with possible adrenal and growth hormone deficiency. Visual acuity was normal bilaterally, with mild papilledema. MRI showed a supradiaphragmatic, transinfundibular-to-retroinfundibular tumor extending posteroinferiorly into the prepontine cistern.^4^ Given its midline stalk origin, and to achieve the close-up, panoramic view needed for precise neurovascular dissection, an endoscopic endonasal approach is employed, using a Storz 4K endoscope with 0° and 30° lenses. Intraoperative visual evoked potential monitoring and neuronavigation are used. Radical resection is planned using a subpial dissection technique to maintain a safe dissection plane and maximize endocrine preservation.^5^

### 2:12 Surgical Procedure.

A binostril, three-hand technique is used. The anterior and posterior ethmoid air cells are opened through both middle meatuses to widen the corridor, and the superolateral recess of the sphenoid sinus is exposed. Bilateral rescue flap incisions are made while preserving the septal cartilage, exposing the vomer bone. To widen the anterior sphenoid wall opening, the palatovaginal canal is bluntly exposed, and its contents are transected. The superior septal mucosa is gently elevated subperiosteally to preserve the olfactory filaments.^6^

### 2:50 Skull Base Drilling.

From here, the neurosurgeon drives the endoscope. The anterior wall of the sphenoid sinus is widely opened, and its mucosa is elevated and preserved as a pedicled flap. As the cancellous bone is removed with a high-speed drill, the cortical bone extending from the anterior skull base to the clivus is exposed. Carefully avoiding injury to the carotid arteries, the remaining bone is thinned to a paper-thin shell. Drilling the inferior sphenoid wall improves inferior maneuverability. After wide bony removal, the thinned bone is carefully peeled away, exposing the dura from the anterior skull base to the sella.

### 3:30 Dural Opening.

The dura is first opened horizontally over the sellar region and at the superior margins of the optic canals, connected by a midline vertical cut, and the dural flaps are reflected laterally in a French-door fashion. The tumor-expanded pituitary stalk is exposed, with the tumor extending from the retrosellar region into the prepontine space. As the arachnoid is elevated cranially, the pial surface of the stalk is exposed, with longitudinal portal vessels running along the anterior pia. The tumor is circumferentially enveloped by the pial layer of the stalk.

### 4:10 Pituitary Transposition.

An intradural pituitary hemitransposition secures adequate working space.^7^ The left side of the pituitary gland is incised, allowing lateral mobilization and creating the operative and visual axes. At the left lateral aspect of the stalk, the transition zone where the pial layer becomes attenuated is identified, serving as the entry point for subpial dissection.

### 4:37 Subpial Dissection.

Dissection begins in the plane between the pia and the tumor, incising the pia vertically at its thinnest portion between the longitudinal portal vessel streams to create the entry point. The tumor is circumferentially enveloped by the pial layer, which thins posteriorly and inferiorly, a transinfundibular-to-retroinfundibular growth pattern. Alternating dissection with internal debulking exposes new planes and avoids blind traction. Subpial dissection continues bilaterally, preserving the surrounding pia, and superiorly separates the tumor from the hypothalamic layer around the infundibular recess. Firm portions are internally debulked with an ultrasonic aspirator. Throughout, the pia is kept intact and the tumor gently separated. Superiorly, the tumor protrudes into the third ventricle and adheres to the ventricular wall,^8^ whose ependymal layer transitions caudally into the thin pial layer of the stalk. Because less adhesion is expected superiorly, the normal ventricular wall plane is identified first and followed caudally. A malleable ring curette reaches even the uppermost part of the tumor. The tumor is separated from the ventricular wall under close-up visualization, with meticulous care to avoid hypothalamic injury. From the hypothalamus to the infundibular recess, dissection follows the ependymal layer; working cranial to caudal helps identify the proper plane and avoid hypothalamic injury.^9^ Around the infundibular recess, the thin pial membrane is preserved; with slight retraction, the stretched pia is returned to its native position for atraumatic dissection. Dissection advances along the tumor capsule while preserving the pial capillaries under direct vision. Even the left posterior portion extending into the prepontine space remains covered by a thin pial layer. Laterally and caudally, the pia is preserved as the outer layer, allowing gentle removal without manipulating the cranial nerves or perforating arteries. The tumor is completely removed. A 5-mg injection of indocyanine green confirms good perfusion of the pituitary stalk, gland, superior hypophyseal arteries, and perforators. The continuous hypothalamic layer from the third ventricle to the infundibular recess is preserved, and the caudal tumor is also removed with the pia intact.

### 7:47 Skull Base Reconstruction.

Multilayer skull base reconstruction is performed to prevent postoperative cerebrospinal fluid leakage.^10^ A free inlay fascia lata graft is sutured to the dural edges with 7-0 Prolene at five points, and the reflected dural flaps are closed in a French-door fashion. The absence of a cerebrospinal fluid leak is confirmed with the Valsalva maneuver. A fascia lata graft with attached fat is then placed and covered with a pedicled sphenoid sinus mucosal flap, and absorbable packing is placed in the nasal cavity to prevent adhesions.

### 8:22 Postoperative Outcomes.

Postoperative MRI confirms gross-total resection. No cerebrospinal fluid leak or epistaxis is observed, and visual function remains unchanged. Stimulation testing showed preserved adrenal function, with mild impairment of the thyroid-stimulating hormone and growth hormone axes and mild diabetes insipidus. Hydrocortisone replacement was discontinued 3 months after surgery. There was no significant weight gain, and the patient returned to kindergarten. Histology confirmed adamantinomatous craniopharyngioma, with no recurrence at 9 months.

### 8:58 Discussion.

The schematic on the left illustrates the tumor’s growth pattern and its relationship to the surrounding layers. The tumor arises from the craniopharyngeal duct and expands while preserving the pial layer of the pituitary stalk, with a thin pial layer assumed to remain on its surface even as it enlarges. By maintaining this layer and dissecting between the pia and the tumor, resection avoids direct manipulation of surrounding neurovascular structures while preserving the pituitary stalk. Our group previously described the vertical infundibular split with subpial excavation technique for transinfundibular craniopharyngiomas;^5^ here, the same concept was applied to a retroinfundibular tumor with supradiaphragmatic extension. Because a continuous pial layer is thought to remain regardless of growth pattern, this technique may be applicable to various suprasellar craniopharyngiomas.

### 9:55 Conclusions.

In conclusion, the subpial dissection technique provides a reliable surgical strategy for endoscopic endonasal surgery of craniopharyngiomas with supradiaphragmatic extension, enabling radical tumor removal while minimizing risk to surrounding structures and preserving the hypothalamus, pituitary stalk, and endocrine function.

## Acknowledgments

Claude (Opus 4.8; Anthropic) was used for minor language and readability edits to portions of the manuscript.

## Disclosures

Dr. Akutsu reported grants from Elekta outside the submitted work.

## Author Contributions

Primary surgeon: Akutsu. Assistant surgeon: Hongo, Morinaga, Shibao, Oshida, Kashiwagi, Tsunemi. Editing and drafting the video and abstract: Akutsu, Hongo, Nakayama, Yamamoto. Critically revising the work: Akutsu, Hongo, Shibao, Nakayama, Yamamoto. Reviewed submitted version of the work: Akutsu, Hongo, Shibao, Iwamoto, Oshida, Nakayama, Yamamoto. Approved the final version of the work on behalf of all authors: Akutsu. Supervision: Akutsu, Nakayama.

## Supplemental Information

### Patient Informed Consent

The necessary patient informed consent was obtained in this study.
